# Incremental Benefits of Male HPV Vaccination: Accounting for Inequality in Population Uptake

**DOI:** 10.1371/journal.pone.0101048

**Published:** 2014-08-04

**Authors:** Megan A. Smith, Karen Canfell

**Affiliations:** 1 School of Public Health, The University of Sydney, Sydney, Australia; 2 Prince of Wales Clinical School, UNSW Australia, Sydney, Australia; Public Health England, United Kingdom

## Abstract

**Background:**

Vaccines against HPV16/18 are approved for use in females and males but most countries currently have female-only programs. Cultural and geographic factors associated with HPV vaccine uptake might also influence sexual partner choice; this might impact post-vaccination outcomes. Our aims were to examine the population-level impact of adding males to HPV vaccination programs if factors influencing vaccine uptake also influence partner choice, and additionally to quantify how this changes the post-vaccination distribution of disease between subgroups, using incident infections as the outcome measure.

**Methods:**

A dynamic model simulated vaccination of pre-adolescents in two scenarios: 1) vaccine uptake was correlated with factors which also affect sexual partner choice (“correlated”); 2) vaccine uptake was unrelated to these factors (“unrelated”). Coverage and degree of heterogeneity in uptake were informed by observed data from Australia and the USA. Population impact was examined via the effect on incident HPV16 infections. The rate ratio for post-vaccination incident HPV16 in the lowest compared to the highest coverage subgroup (RR_L_) was calculated to quantify between-group differences in outcomes.

**Results:**

The population-level incremental impact of adding males was lower if vaccine uptake was “correlated”, however the difference in population-level impact was extremely small (<1%) in the Australia and USA scenarios, even under the conservative and extreme assumption that subgroups according to coverage did not mix at all sexually. At the subgroup level, “correlated” female-only vaccination resulted in RR_L_ = 1.9 (Australia) and 1.5 (USA) in females, and RR_L_ = 1.5 and 1.3 in males. “Correlated” both-sex vaccination increased RR_L_ to 4.2 and 2.1 in females and 3.9 and 2.0 in males in the Australia and USA scenarios respectively.

**Conclusions:**

The population-level incremental impact of male vaccination is unlikely to be substantially impacted by feasible levels of heterogeneity in uptake. However, these findings emphasize the continuing importance of prioritizing high coverage across all groups in HPV vaccination programs in terms of achieving equality of outcomes.

## Introduction

Vaccination of pre-adolescent females against human papillomavirus (HPV) has been recommended or included in publicly funded programs in many developed countries. To date, four counties have also recommended that pre-adolescent males also be vaccinated – Australia, Austria, Canada and the United States [1]–[4]. Thus far only Australia has implemented a national publicly funded vaccination program which includes boys (from 2013), and Austria has announced a publicly funded program including boys will commence from 2014 [5]. Many other developed countries are still to make decisions around whether or not to fund or recommend male vaccination.

Previous modelling studies have shown that the incremental impact of vaccinating males depends on uptake in females, with the incremental benefit (and thus cost-effectiveness) decreasing with increasing female coverage [6]–[8], and that increasing coverage further in females can be more effective and cost-effective than including males in vaccination programs [8]–[10]. The underlying mechanism is that increasing female coverage will increase the proportion of heterosexual partnerships where at least one partner is vaccinated [8], and so increase indirect protection (herd effects). Indirect protection plays an important role in the incremental effectiveness of male vaccination compared to female-only programs, because as female coverage increases, so too does indirect protection for males and unvaccinated females; therefore the extent to which vaccinating males can offer additional protection becomes progressively smaller. However, if factors influencing vaccine uptake also influence sexual partner choice, this would potentially affect the extent of indirect protection, and as a result alter the incremental impact of male vaccination. To date, the large majority of modelling studies have assumed that choice of sexual partner and sexual behavior are not correlated with vaccination uptake – that is, that any factors which may be associated with vaccine uptake are not also associated with aspects of sexual behavior. However, this assumption is unlikely to hold in practice since sociodemographic, cultural and geographic factors are all likely to have a substantial bearing on both sexual partner choice and vaccination uptake in some settings. Such associations, may be, but are not necessarily, mediated by levels of sexual activity. For example, geographic factors are likely to impact on partner choice across different behavioral groups and also are likely to play a role in vaccine coverage, particularly where vaccination delivery is coordinated at a regional or state level.

Two recent systematic reviews of factors associated with vaccine uptake in teenage girls, based primarily on studies conducted in the USA, identified race, area of residence, cost/insurance status, physician's recommendation, completion of other recommended vaccinations, and parental concerns about vaccine safety as important factors [11], [12]. These factors have also been identified in more recent studies [13]–[18]. Factors associated with uptake may vary depending on the method of delivery (school- versus primary care/clinic-based), funding model (publicly-funded versus insurance- or private-payer) and between countries. For example, a recent study from Canada found that socioeconomic status was associated with uptake where the vaccine delivery was clinic-based, but not when it was school-based [19]. This is also supported by data from two settings with school-based publicly-funded programs which found little effect of socioeconomic status on uptake [20], [21]; in contrast, in the United States public funding is limited and insurance status appears to affect uptake [11]–[13]. The extent to which ethnicity is associated with uptake has varied between studies [11], [12], and the underlying reason behind any identified associations with ethnicity may differ between countries. For example, a recent review for the United Kingdom found some evidence of variation in acceptability and uptake by ethnicity, but that this may in practice have been a proxy for religion [22]. Data from the USA suggest that non-religious factors underpin the association with ethnicity, as parental intention to vaccinate their child did not vary by ethnicity, whereas uptake did [18]. Since male HPV vaccination has not yet been widely adopted internationally, studies looking at factors associated with uptake in young males have been very limited. However, associations are likely to be similar to those observed for females [23], [24], and factors such as race, cost/insurance status, physician's recommendation and completion of other recommended vaccinations have also emerged in two recent studies of uptake in young males in the USA [14], [25]. Parents whose daughters had already received the HPV vaccine were more willing for their sons to receive it [26], [27] and gender of child is not generally associated with acceptability to parents [28], [29]. Therefore, it is plausible that factors influencing uptake in females are likely to also influence uptake in males.

The simplifying assumption that factors influencing vaccine uptake are not also associated with aspects of sexual behavior may have impacted the accuracy of prior estimates of both the effectiveness of female vaccination and the incremental benefit of male vaccination. No prior study has performed a comprehensive evaluation of the impact of correlation between vaccine uptake and sexual behaviour (whether driven by cultural, geographic or other factors) on estimated vaccination outcomes. Two prior Canadian studies have assessed the direct impact of differential vaccination uptake in sexual behavior subgroups (i.e. subgroups defined according to rates of partner change and age of onset of sexual activity) for female [30] and both-sex [31] vaccination. Malagón *et al* [31] found that disparities in uptake would potentially reduce population-level effectiveness if uptake was lower in more sexually active groups, but that this effect decreased at higher levels of vaccine coverage. However, no studies to date have considered the impact of factors influencing partner choice other than, as above, the direct impact of differential coverage according to absolute level of sexual activity (number of sexual partnerships), and nor has any study assessed the extent to which including males affects the differences in outcomes between groups in a program with differences in female uptake. Additionally, quantifying between-group differences has rarely been done in modelling studies; these have generally focused on population-level effectiveness and cost-effectiveness, because these are critical factors in policy decisions. In this regard, however, a highly influential report to the WHO has recommended that health equity impact be routinely assessed for proposed policies [32].

The aims of the current study were, therefore, to perform a generalized assessment of the impact of a correlation between factors influencing vaccine uptake and choice of sexual partner; where such a correlation could be underpinned by a number of plausible mechanisms driving changes in both outcomes, including geographic, socioeconomic and cultural elements. We examined the impact at the population level of adding males to HPV vaccination programs if factors influencing vaccine uptake also influence partner choice. Additionally, we aimed to quantify how these specific kinds of differential uptake may change the distribution of disease between subgroups after vaccination, using incident infections as the outcome measure. Inequalities between groups here are described, and represent between-group differences in either vaccination uptake or infection outcomes after vaccination. However it should be noted that inequality is not by itself directly interpretable as “inequity”, whichis often seen as inequality which is avoidable and unfair, [33].

## Methods

### Model description

A dynamic model of HPV16 was used to simulate sexual behavior and HPV vaccination, transmission and natural history in a population. This model has been previously described in detail [7], [34], [35]. Briefly, the model simulates the potential transmission of HPV during heterosexual partnerships in a population which is closed and stratified by sex, age and level of sexual activity. Model parameters were obtained via literature reviews and fitting to observed data [7], [34].

Simulations were performed under various conditions, to reflect either scenarios where vaccine uptake within the population was correlated with factors which also affect choice of sexual partners (“correlated”), or where uptake was unrelated to any of these factors (“unrelated”). In the “correlated” scenario, subgroups were simulated in separate closed models which did not interact sexually, to represent mixing which is assortative with respect to factors correlated with vaccine uptake. Different levels of vaccine uptake were applied in different subgroups, but all other model parameters were consistent between subgroup populations (for example sexual behavior, including the proportion of the population in different sexual behavior strata, and all aspects of vaccine efficacy and HPV natural history). In the corresponding “unrelated” scenario the equivalent population-level vaccine uptake, which was derived by weighting the subgroup coverage rates by the respective sizes of the subgroups, was applied across the population.

### Vaccination coverage scenarios

This analysis does not explicitly model any specific factors associated with vaccine uptake; rather, this was intended to be an exploratory example from which broad conclusions could be drawn. This was done because it appeared that factors associated with vaccine uptake (encompassing both the aspects of initiation and completion of the three-dose series) vary in different settings.

**Figure 1:**
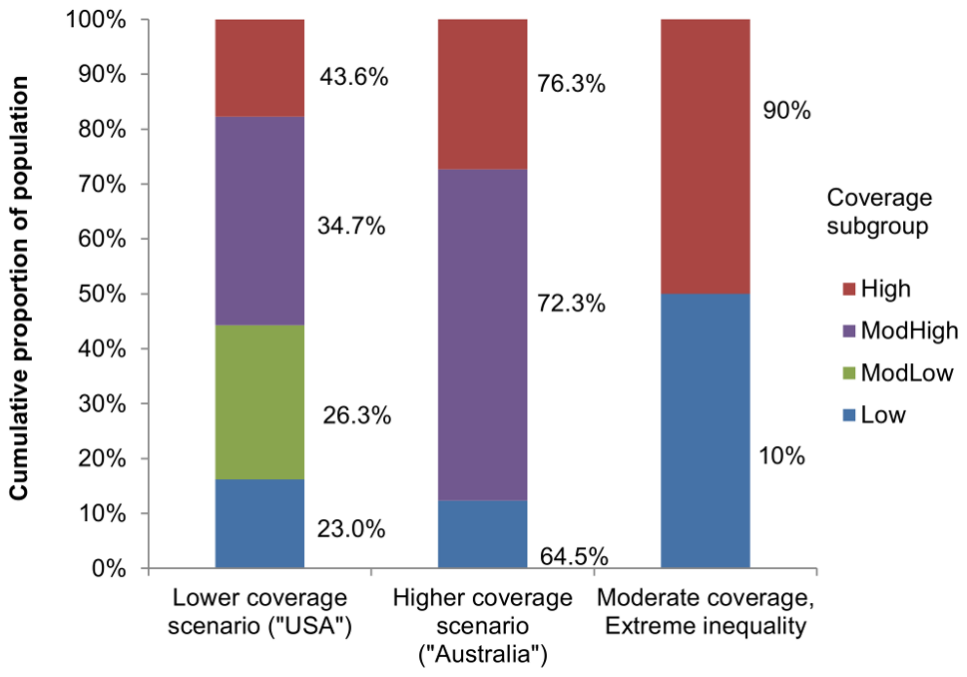
Subgroup size and vaccine uptake in modelled coverage scenarios. Values next to bar represent coverage in that subgroup; bar height represents subgroup size.

However, the scenarios chosen were informed by data from actual HPV vaccination experiences. Three population coverage scenarios were considered: “Australia” (72% overall); “USA” (32% overall); and a hypothetical exploratory scenario with intermediate coverage (50% overall) where the “correlated” variant explored “extreme inequality”. The “Australia” coverage scenario was based on observed vaccine uptake in Australia for girls aged 15 years in 2009 (who were offered vaccination in 2007 and 2008). Three subgroups were formed, with their size and vaccine coverage based on three-dose uptake within the different states, grouped based on broad coverage levels [36], [37]. Uptake in the subgroups ranged from 64.5% to 76.3%. The “USA” population coverage scenario was based on observed vaccine uptake for females in the USA who were aged 13 to 17 years in 2010. Four subgroups were formed, with their size and vaccine coverage likewise based on three-dose uptake within the different states grouped based on broad coverage levels [38], [39]. Uptake in the subgroups ranged from 23.0% to 43.6%. In each coverage scenario, the equivalent overall population coverage used in the “unrelated” variant of the scenario was the weighted average of three-dose coverage across all subgroups. Detailed information on coverage assumptions in each case is presented in Figure 1 and Table S1 in File S1.

In each case, we compared results from a female-only program to those from a both-sex program. We also examined the extent to which including males in a program altered the distribution of disease between groups in the population after vaccination. All scenarios assumed that vaccination was delivered at age 12 years. In the baseline analysis, we assumed equal coverage in males and females, but assessed lower coverage in males relative to females in a sensitivity analysis.

### Assessing outcomes and quantifying between-group differences in outcomes

The age-standardized rate (ASR) of incident HPV16 infections at post-vaccination equilibrium (i.e. several decades after the implementation of vaccination) was used as the outcome measure and a measure of post-vaccination disease burden. Outcomes from the model were examined at both the population level and at the subgroup level. Outcomes at the population level were calculated for the “correlated” uptake scenario by weighting the outcomes from the individual subgroups according to their relative size in the population. Comparisons were then made with outcomes from the corresponding “unrelated” uptake scenario.

For comparison of outcomes in different subgroups, two approaches were used. In the first, the cumulative proportion of the population represented by each subgroup was plotted against the cumulative proportion of the burden of disease experienced by that subgroup (pseudo Lorenz curve). As previously, incident HPV16 infections at post-vaccination equilibrium were used as the measure of post-vaccination disease burden; these infection rates were assumed to be equivalent in all subgroups (but not in all sexual behavioral, sex and age strata within subgroups) prior to vaccination. In a population where outcomes are equal across the subgroups, the plotted pseudo Lorenz curve would be a diagonal line; the further away this plot is from the diagonal line of equality, the greater the degree of inequality in outcomes. The distance between the ideal diagonal line and the situation which arises in each scenario can be quantified by calculating a pseudo Gini coefficient, a measure of dispersion in outcomes between different groups, which is equal to twice the area between the plotted pseudo Lorenz curve and the ideal diagonal [40]. A coefficient of zero represents perfect equality, whereas the closer the value is to the theoretical maximum, the more unequal outcomes are in subgroups. Since outcomes are considered at the level of a subgroup (rather than the individual level), the theoretical maximum of the coefficient will depend on the subgroup sizes. Here the theoretical maximum was calculated to represent the most unequal situation possible, which was assumed to occur if all disease occurred in the subgroup with the lowest vaccine uptake. The Lorenz curve and Gini coefficient are well recognized measures of dispersion, and the pseudo Gini coefficient has been extensively used in measuring health inequalities [40]–[42].

The second approach to comparing outcomes between subgroups calculated the rate ratio (RR_L_) as the ratio of the post-vaccination equilibrium age-standardized rate of incident HPV16 infections in the subgroup with the lowest vaccine coverage relative to that in the subgroup with the highest coverage. We additionally used a rate ratio because this is a commonly understood concept in health; however it is a less desirable measure of inequality, as it does not take into account the whole population, nor the sizes of the subgroups with differing outcomes [40].

### Sensitivity analysis

Parameters which were found to have the most influence on outcomes in prior work [7], including vaccine coverage and aspects of vaccine efficacy, were varied during sensitivity analysis. Natural history assumptions were not varied, as previous work showed that using alternative model parameter sets which had been fitted to HPV prevalence had little impact on predicted vaccination outcomes [7]. Details of the assumptions used for sensitivity analysis are available in the accompanying Supplementary Material (see *Sensitivity Analyses* in File S1). Additional sensitivity analyses varied the extent of heterogeneity in vaccine uptake in the “Australia” and “USA” coverage scenarios, in either both sexes or males only (see *Exploratory Analyses* in File S1).

## Results

### Population level outcomes

**Figure 2:**
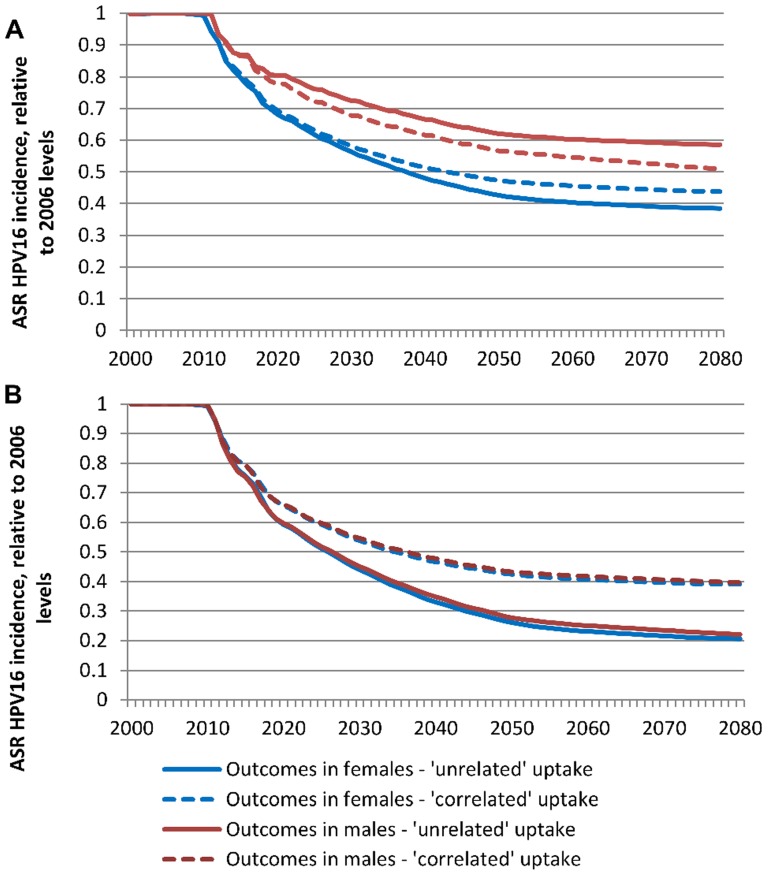
Impact of heterogeneity in vaccine uptake on population level outcomes. (A) Female-only program (50% overall coverage, extreme inequality). (B) Both sex program (50% overall coverage, extreme inequality). “Correlated” uptake refers to a situation where vaccine uptake within the population is correlated with factors which also affect choice of sexual partners. “Unrelated” uptake refers to a situation where vaccine uptake is unrelated to any of these factors. Vaccination was assumed to commence in 2007.

In a female-only program with 50% overall coverage, the predicted long-term relative reductions in female and male HPV infections are 56% and 49% for “correlated” uptake under assumptions of ‘extreme inequality’, compared to 62% and 41% for “unrelated” uptake, respectively (Figure 2a). If males were also vaccinated at the same coverage as females, the predicted reductions in female and male infections are 61% and 60% for “correlated” uptake with extreme inequality, compared to 79% and 78% for “unrelated” uptake, respectively (Figure 2b). Similar effects were seen in the “Australia” (higher) and “USA” (lower) coverage scenarios, where the heterogeneity was less pronounced, but in these scenarios the effects were extremely small (<1% difference in population impact) (Table 1). Generally “correlated” uptake resulted in a lower vaccination effectiveness at the population level, except that female-only programs with “correlated” uptake resulted in better outcomes in males (but not in females), than female-only programs with equivalent “unrelated” uptake. The extent to which this occurred varied depending on the degree of heterogeneity in uptake between subgroups, but can be explained by considering the implications in the simple “extreme inequality” scenario. In this example, the assumption that coverage occurred in equal-sized subgroups, one with higher (90%) and one with lower (10%) coverage resulted in a net loss of benefit for females, because the extent of indirect (herd) protection was lower in both of the subgroups than in the scenario with moderate (50%) coverage in females which was uniform across the population. Females in the lower coverage subgroup did not experience a large herd effect because coverage was very low; and within the higher coverage subgroup, there was less room for improvement in outcomes due to herd effects, since coverage, and impact, was already very high for females. In contrast, males who partnered within the subgroup of females with very high uptake experienced strong herd effects. As the benefits to males from a female-only program do not scale linearly with coverage, and appear to accelerate as coverage in females increases [43], [44], the gains in this subgroup of males outweighed the loss for males in the lower coverage subgroup.

**Table 1:** Summary of main results, by sex, coverage scenario and program type.

	POPULATION LEVEL OUTCOMES (%reduction in incident HPV16 infections at equilibrium)				EQUALITY OUTCOMES			
	“Unrelated”^a^		“Correlated”^a^		Pseudo Gini coefficient^b^		RR_L_ c	
Scenario	Females	Males	Females	Males	Females	Males	Females	Males
Higher coverage (“Australia”)								
Female-only	84.2%	65.8%	84.1%	65.9%	0.0936	0.0578	1.9	1.5
* Both sexes*	*96.4%*	*95.7%*	*96.2%*	*95.6%*	*0.2205*	*0.2086*	*4.2*	*3.9*
Lower coverage (“USA”)								
Female-only	40.8%	24.9%	40.6%	25.0%	0.0771	0.0439	1.5	1.3
* Both sexes*	*57.4%*	*56.0%*	*56.7%*	*55.4%*	*0.1258*	*0.1200*	*2.1*	*2.0*
Moderate (50%) coverage; extreme inequality								
Female-only	61.5%	41.4%	56.2%	49.1%	0.4696	0.4002	31.9	9.0
* Both sexes*	*79.4%*	*77.9%*	*60.9%*	*60.4%*	*0.4997*	*0.4996*	*3,317.2*	*2,466.7*

a “Correlated” uptake refers to a situation where vaccine uptake within the population is correlated with factors which also affect choice of sexual partners. “Unrelated” uptake refers to a situation where vaccine uptake is unrelated to any of these factors.

b A pseudo Gini coefficient closer to zero represents more equal outcomes between subgroups; a pseudo Gini coefficient closer to the theoretical maximum represents more unequal outcomes between subgroups. Theoretical maxima for pseudo Gini coefficients are 0.8766 (“Australia” scenario), 0.8378 (“USA” scenario) and 0.5 (“extreme inequality” scenario).

c RR_L_ is the risk experienced by the subgroup with the lowest vaccine coverage relative to that in the subgroup with the highest vaccine coverage, obtained by dividing the age-standardised rate of incident HPV16 infections in the subgroup with the lowest vaccine coverage by the corresponding rate in the subgroup with the highest vaccine coverage.

### Subgroup level outcomes

**Figure 3:**
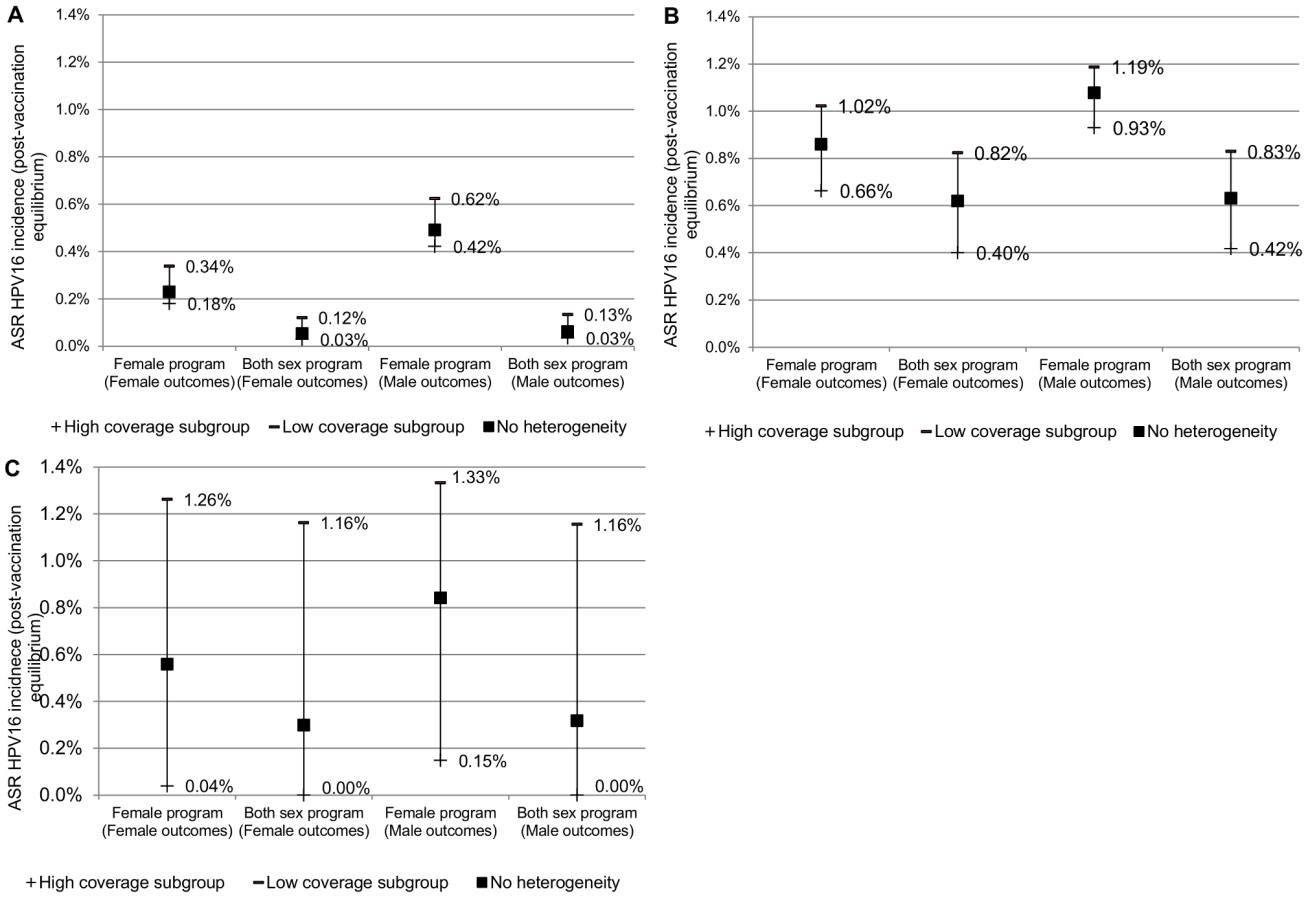
Impact of heterogeneity of vaccine uptake on subgroup outcomes. (A) Higher population coverage (“Australia”; 72.4% overall). (B) Lower population coverage (“USA”; 32.1% overall). (C) 50% overall coverage, extreme inequality.

Compared to female-only vaccination, including males in the vaccination program improved outcomes in all subgroups, as the absolute risk of HPV16 infection reduced in all subgroups and in both sexes (Figure 3).

**Figure 4:**
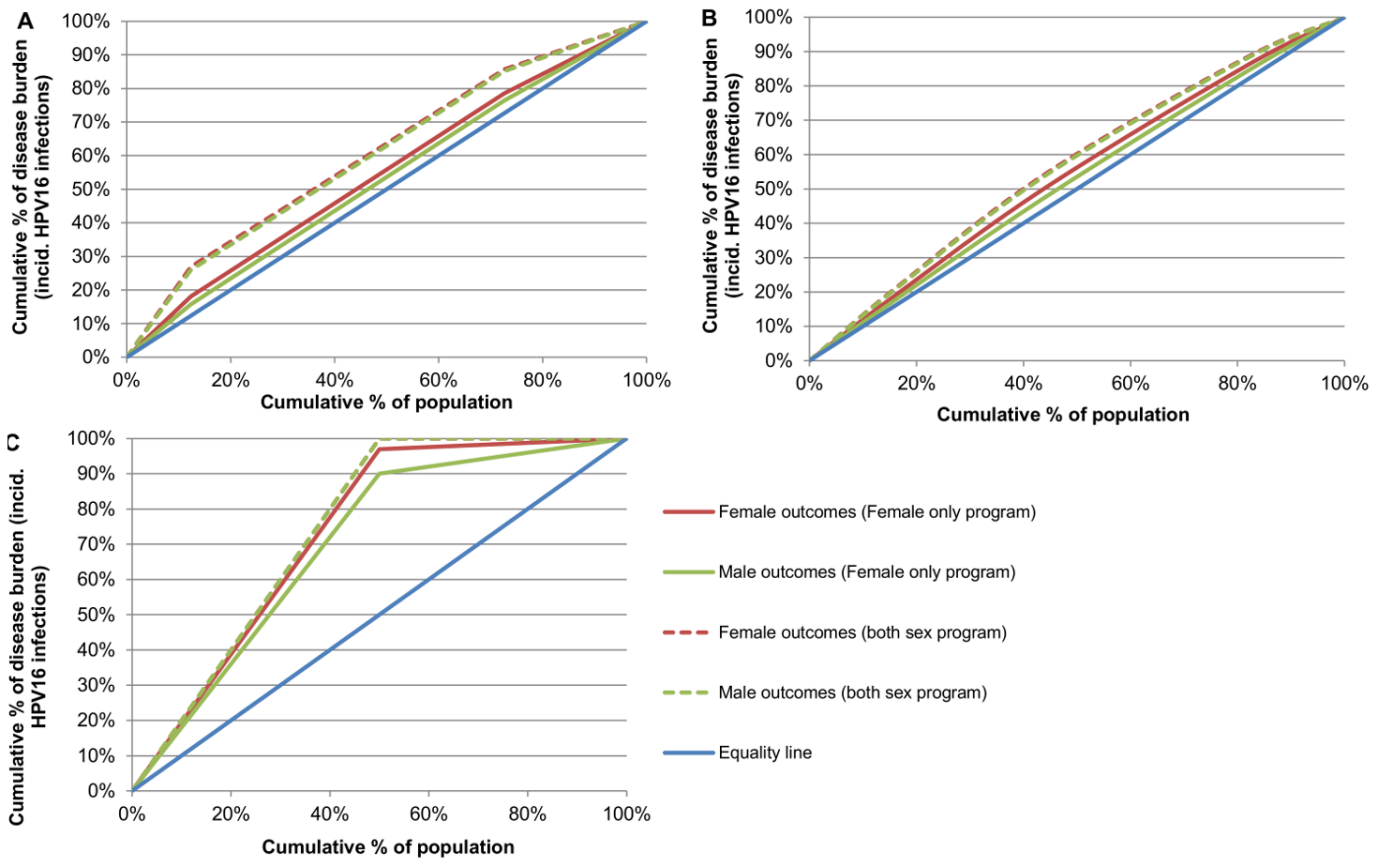
Distribution of disease outcomes (incident HPV16 infections) across subgroups (pseudo Lorenz curve). (A) Higher population coverage (“Australia”; 72.4% overall). (B) Lower population coverage (“USA”; 32.1% overall). (C) 50% overall coverage, extreme inequality. Comparison of the proportion of disease borne by each subgroup with the group's size. The diagonal line represents a situation where there are no inequalities in outcomes between subgroups; the further away a plot of outcomes is from this equality line, the more unequal outcomes are in that scenario. The pseudo Gini coefficient represents twice the area between the pseudo Lorenz curve and the equality line.

**Figure 5:**
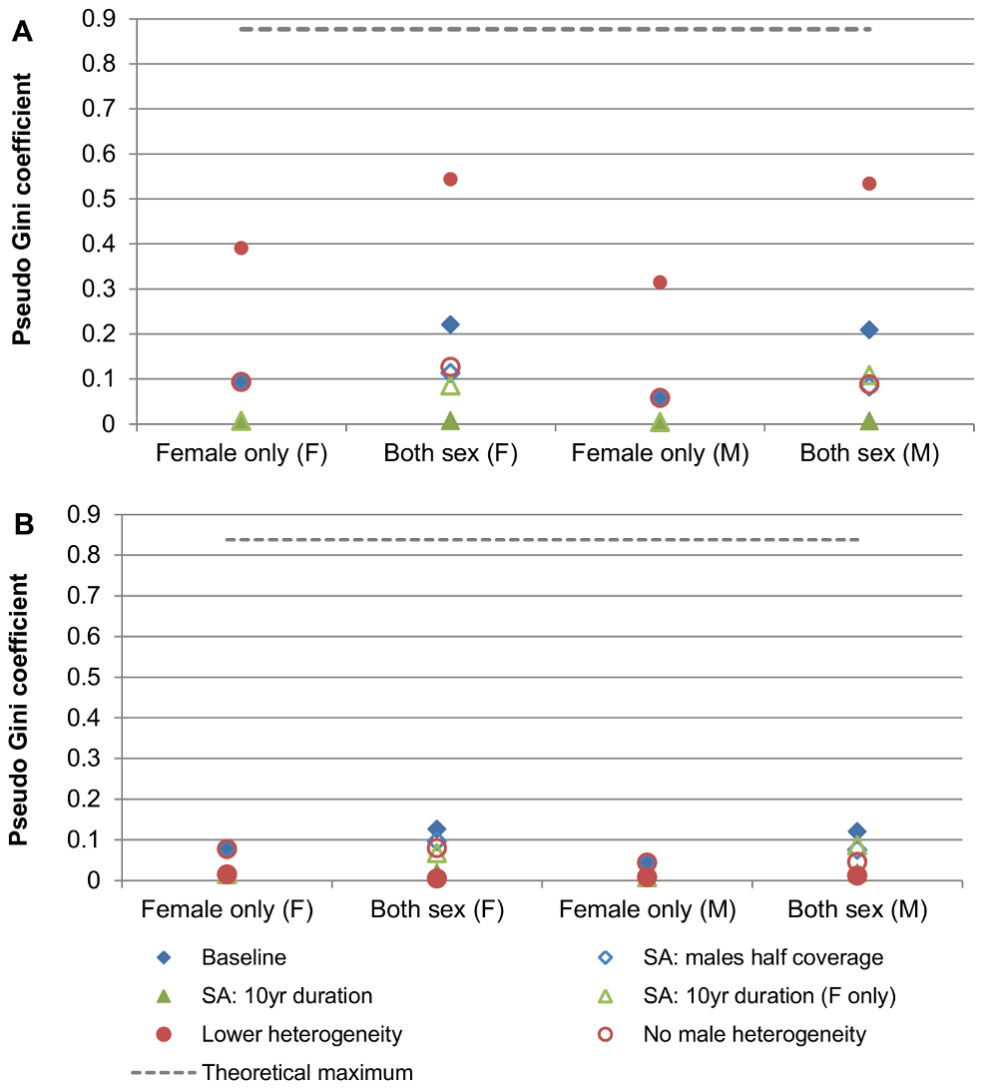
Impact of varying model assumptions on inequality of outcomes. (A) Higher population coverage (“Australia”; 72.4% overall). (B) Lower population coverage (“USA”; 32.1% overall). A higher value of the pseudo Gini coefficient represents more unequal outcomes. (F) denotes the value for the pseudo Gini coefficient relating to outcomes in females; (M) denotes the value for the pseudo Gini coefficient relating to outcomes in males. SA =  sensitivity analysis. * Switched heterogeneity: Higher heterogeneity used for Australia scenario ((equivalent to heterogeneity in main USA scenario); Lower heterogeneity used for USA scenario (equivalent to heterogeneity in main Australia scenario).

As expected, female-only vaccination with “correlated” vaccine uptake resulted in differential outcomes between subgroups, and this was more pronounced for outcomes in females than in males (Figure 4). However, in the higher and lower coverage scenarios examined, the differences in outcomes between subgroups were comparatively small (Table 1). Including males in the program further increased the differences between subgroups after vaccination, and the level of inequality became similar for males and females (Figure 4). The absolute value of the pseudo Gini coefficient increased for males and females when males were included in programs, indicating outcomes had become more unequal between the subgroups, both in females and males (Table 1). The extent of this varied, however, and was more pronounced in the higher “Australia” coverage scenario than the lower “USA” coverage scenario, as in the higher coverage scenario the post-vaccination incidence of disease in the subgroup with highest coverage was extremely small (Figure 5). In the lower coverage scenario, the pseudo Gini coefficient increased from 0.0771 to 0.1258 for females and from 0.0439 to 0.1200 for males when males were also vaccinated (theoretical maximum for four subgroups modelled: 0.8378; Table 1). In the higher coverage scenario, the pseudo Gini coefficient increased from 0.0936 to 0.2205 for females and from 0.0578 to 0.2086 for males when males were vaccinated (theoretical maximum for three subgroups modelled: 0.8766). In the scenario with moderate coverage but extreme inequality, the pseudo Gini coefficient increased from 0.4696 to 0.4997 for females and 0.4002 to 0.4996 for males when males were vaccinated (theoretical maximum for two subgroups modelled: 0.5).

Using an alternative measure of inequality, the rate ratio, produced similar results; that is, that “correlated” female vaccination led to differential subgroup outcomes in females and also to a lesser extent in males, and that including males increased the between-group differences in outcomes. Female-only vaccination with “correlated” vaccine uptake resulted in RR_L_ = 1.9 (“Australia” scenario),1.5 (“USA” scenario) or 31.9 (extreme inequality scenario) in females, and RR_L_ = 1.5,1.3 and 9.0 in these scenarios in males. Both-sex vaccination with “correlated” uptake increased RR_L_ to 4.2, 2.1 and 3,317 in females and 3.9, 2.0 and 2,467 in males in programs with higher, lower, or extreme inequality in coverage respectively (Table 1).

### Sensitivity analysis

The predicted reductions in female and male infections, and the incremental benefit achieved by adding males, remained very similar at the population level for “correlated” and “unrelated” uptake in the “Australia” and “USA” scenarios during sensitivity analysis. The difference in the predicted percentage reduction in infections at the population-level from a program with “unrelated” versus “correlated” uptake was less than one percentage point in almost all cases. The exception was a version of the “Australia” scenario where heterogeneity in vaccine uptake was greater (equivalent to that observed in USA data), although the difference was still quite small (less than five percentage points). Sensitivity analysis demonstrated that adding males to female-only programs consistently improved the absolute outcomes in all subgroups, regardless of overall coverage level, or duration of vaccine protection. The incremental benefit of adding males was greatest when vaccine protection was long-lasting in males, but not in females. However, compared to the female-only program, adding males also consistently increased between-group differences in outcomes, although the extent of this effect varied (Figure 5). Scenarios where coverage was lower in males, or duration of vaccine protection was short in males, had less impact on the degree of inequality. However the reason for this was because in practice these programs were less effective over the long term, and so the relatively greater benefits experienced by the subgroups with higher uptake were short-lived and did not increase between-group differences over the long term. Adding males with uniform uptake also had less impact on the degree of inequality, however this was still increased compared to female-only vaccination (Figure 5; see also File S1).

The magnitude of between-group differences in outcomes was influenced both by the extent of inequality in vaccine uptake, and also by the overall population coverage level. For programs with equivalent heterogeneity in vaccine uptake, between-group differences in outcomes were greater in a program with higher coverage than one with lower coverage (Figure 5; also see *Exploratory Analyses* in File S1).

## Discussion

Some factors which have high potential to influence HPV vaccine uptake include geographical location within a country (since programs are often organized at a regional or state level), cultural identity (since programs may be more or less successful at reaching different groups) and sociodemographic factors (since, particularly when vaccination delivery is not school based, sociodemographic factors may affect the accessibility of or decision to be vaccinated) [11], [12], [19], [21], [36], [38], [45]. These factors are also likely to influence the social networks in which people congregate and thus the pool from which potential sexual partners are chosen. The impact of a partial correlation between sexual partner choice and vaccine uptake on vaccination effectiveness has not previously been assessed. We found that inequalities in vaccine uptake which are correlated with factors that also affect choice of sexual partner do not adversely impact the population level effectiveness of vaccination programs, unless the inequalities in uptake are pronounced. Reassuringly, this finding suggests that the estimated cost-effectiveness of both female and male HPV vaccination, based on evaluations which to date have assumed homogenous uptake, is unlikely, except in extreme situations, to be substantially affected by this kind of inequality. Although “correlated” uptake generally resulted in a worse outcome at the population level, the difference was extremely small in most scenarios we explored. The comparatively small differences at the population level can be explained by the comparatively small degree of heterogeneity in uptake in the observed data from Australia and the USA, and thus the comparatively small differences which arose in the probability that at least one partner in a heterosexual relationship was vaccinated. Herd effects are related to the chance that at least one partner in a relationship is vaccinated [8], and the degree of heterogeneity modeled in our “Australia” and “USA” coverage scenarios did not substantially alter the overall probability that at least one partner would be vaccinated in a heterosexual partnership compared to the “unrelated” scenario. Interestingly, female-only programs with “correlated” uptake resulted in better population-level outcomes in males (but worse population-level outcomes in females), than female-only programs with equivalent “unrelated” uptake. In effect, “correlated” uptake in a female-only program appears to shift some of the vaccination benefits from females to males (relative to those which would be achieved by a “unrelated” program), specifically, shifting benefits to the male partners of females in the subgroups with higher coverage.

Our study confirmed that vaccinating males improved outcomes in all subgroups, even if uptake was correlated with factors that also affect choice of sexual partner. However, we also found that in this situation including males in vaccination programs tended to further concentrate disease in particular subgroups and increase between-group differences in outcomes, even if uptake is uniform in males. In these situations, decision-makers may face a trade-off between reducing inequalities versus reducing the levels of disease overall. It would likely then be important to take into account the highly setting-specific factors of who the subgroups are, their relative advantage and disadvantage overall, their other risk factors, and the potential alternative uses of resources, and whether the inequality is avoidable or not. That is, decision makers would need to take into account whether or not the inequalities represent inequity in a particular setting, given that inequity is often and additionally defined as inequality which is avoidable and unfair [33]. For example if infections were likely to become more concentrated in subgroups that were already disadvantaged, or in females less likely to attend cervical screening and thus at higher risk of cervical cancer, including males may be seen as less favorable than alternative interventions (including, for example, interventions to achieve higher uptake in the disadvantaged subgroups). Conversely, reducing HPV infections further in a particular subgroup could be beneficial if individuals in that group are otherwise disadvantaged, or less likely to be screened. We could not and have not examined inequity *per se* in this generalized assessment, because this would require consideration of setting-specific issues. Setting-specific analyses could incorporate measures which have been proposed to quantify inequity, rather than inequality, such as the concentration index, which could not be used here as they require subgroups to be ordered according to a gradient of socioeconomic status or advantage [40].

We have used the post-vaccination rate of new HPV infections as a proxy for disease risk in different subgroups, since it provides an outcome which is comparable for males and females. The current analysis does not simulate long term outcomes of infections, such as cancer. This is because the risk of developing an HPV-related cancer depends on a range of further factors; in particular cervical cancer risk would be strongly influenced by cervical screening behavior. The specific impact on cervical cancer would also depend on whether variations in uptake are correlated with screening uptake, and the direction of the relationship. For example in the United States, HPV vaccine uptake is higher in areas where screening coverage is higher [46], whereas in New Zealand some ethnic groups with lower screening coverage have higher HPV vaccine uptake [45], [47], We have also only modelled one HPV type, HPV16, in this analysis. HPV16 is associated with the highest risk of oncogenesis in humans and this approach is consistent with other exploratory model-based analyses [10], and done on the basis that analyses for other types would give qualitatively similar results.

A previous study by Malagón *et al* [31] assessed the population-level impact of disparities in vaccine uptake if vaccine uptake differed according to level of sexual activity (i.e. number of partnerships and age at initiation), whereas we performed a complementary assessment of the effects of differences in vaccine uptake according to any factor that influences the choice of specific partner(s). Our results were similar in finding that pronounced heterogeneities in vaccine uptake may affect the population-level impact of HPV vaccination programs. Our finding that including males tended to increase inequality relative to a female-only program differed from the findings of the previous study; however this particular finding from Malagón *et al* [31] was based on a scenario with uniform uptake across subgroups where inequalities were driven by varying herd effects in the subgroups resulting from their different levels of sexual behaviour (since even uniform uptake in females increased inequality). While there were differences in the precise behavioural subgroups modelled and factors associated with vaccine uptake between the two studies, an exploratory analysis suggested that another reason for the different findings was that different measures were used to quantify inequality. One measured the absolute level and distribution of disease between groups, whereas the other measured the relative impact of vaccination. Information about the exploratory analysis and a more detailed discussion of these issues are available in Supplementary Material (see *Exploratory Analyses* in File S1).

An argument in favor of male vaccination is that it would improve equality between males and females. In order to fully explore equality in relation to sex, it would be necessary to consider additional disease endpoints, such as HPV-related cancer, genital warts and recurrent respiratory papillomatosis, to fully capture the differences in the burden of HPV-related disease by sex. While we have not focused on the differences in outcomes in males versus those in females here, several points are worth considering. Firstly, modelling studies have consistently shown that targeting one sex for HPV vaccination is more effective at reducing the overall burden of disease in the population (as well as being more cost-effective) [8]–[10]. Secondly, the burden of HPV-related disease is generally much greater in females than in males, and this is why vaccination of females only (rather than males only) is more effective and cost-effective [10], [48]. Thirdly, the obvious inequality arising from a female-only vaccination program is not in males generally (since their burden of HPV-related disease was lower than that of females prior to vaccination, and is likely to decrease as a result of female vaccination due to herd effects [7], [8], [10], [49]); rather, it will specifically be in men who have sex with men (MSM). MSM have a higher burden of HPV-related disease than other males, particularly anal disease [50], [51], and are less likely to benefit from herd effects from a female-only program [52]. Vaccination of MSM has been shown to be cost-effective in at least one setting, even in early adulthood when targeted vaccination may be more feasible [53], [54]. This is an important topic for further research in settings with female-only vaccination, although local factors such as the disease burden and HIV prevalence will be influential [53].

In our model, the subgroups we considered were assumed not to interact sexually. This did not mean that vaccinated individuals only partnered with other vaccinated individuals (or unvaccinated only with unvaccinated), or that sexual behavior was homogenous within the subgroups; instead it implied that subgroups with varying vaccine uptake (potentially representing geographic, cultural or socioeconomic subgroups) did not interact sexually. In reality, subgroups are not closed, however our modelling assumption allowed us to explore the opposite extreme assumption to that previous models of male vaccination have generally made, which is that uptake is uniform, or equivalently that factors associated with vaccine uptake are unrelated to factors associated with choice of partner. Where inequalities in uptake were not very great, population-level effectiveness was very similar to the “unrelated” variant, even under our extreme and conservative simplifying assumption that subgroups did not intermix, and so this result should apply to the more realistic situation where subgroups are not completely closed. However, given that the assumption of closed subgroups was an extreme assumption for our main population-level question, between-group differences will be more muted in the more realistic situation where subgroups are not closed.

In conclusion, this analysis suggests that the incremental impact (and therefore the cost-effectiveness) of female-only or male vaccination will not be substantially impacted by heterogeneity in population uptake unless there is pronounced inequality in vaccine uptake that is associated with factors influencing partner choice. Outcomes in all subgroups can be improved by vaccinating males as well as females, but including males could increase the degree of inequality in outcomes between population subgroups, whether the variations in uptake between subgroups carry through to males or not. Therefore, reducing inequalities in vaccine uptake remains an important goal, and its importance is not diminished by extending female-only vaccination programs to include males, since including males may exacerbate rather than mitigate between-group differences in outcomes. In general terms these findings emphasize the continuing importance of achieving high coverage in all geographic, cultural and socioeconomic groups in HPV vaccination programs.

## Supporting Information

File S1 **Supporting text and tables.** **Table S1.** Subgroup size and vaccine uptake in coverage scenarios modelled. **Table S2.** Summary of main results in exploratory analysis, by sex, coverage scenario and program type. **Additional Analyses Performed - Sensitivity Analyses and Exploratory Analyses.**
(DOCX)
