# Supplementary material for: Incremental Benefits of Male HPV Vaccination: Accounting for Inequality in Population Uptake
Source: PLoS One. 2014 Aug 4;9(8):e101048. doi: 10.1371/journal.pone.0101048 (PMC4121069; doi:10.1371/journal.pone.0101048)
Supplement: File S1 — Supporting text and tables. Table S1. Subgroup size and vaccine uptake in coverage scenarios modelled. Table S2. Summary of main results in exploratory analysis, by sex, coverage scenario and program type. Additional Analyses Performed - Sensitivity Analyses and Exploratory Analyses. (DOCX) [file pone.0101048.s001.docx]

Supplementary data relating to the manuscript:

Incremental benefits of male HPV vaccination: Accounting for inequality in population uptake

**AUTHORS & AFFILIATIONS**

Megan A Smith MPH BE

PhD student

School of Public Health, University of Sydney, Sydney, NSW, Australia

Program Manager – Cancer Modelling

Prince of Wales Clinical School, UNSW Australia, Sydney, NSW, Australia

Karen Canfell DPhil

Associate Professor

Prince of Wales Clinical School, UNSW Australia, Sydney, NSW, Australia

**Corresponding author:**

Megan Smith

Program Manager Cancer Modelling

Email: megan.smith@unsw.edu.au

Prince of Wales Clinical School,

Lowy Cancer Research Centre C25

UNSW

Sydney NSW 2052

Australia

Tel: + 61 2 9385 1393

Fax: +61 2 985 1430

Megan A Smith1,2* and Karen Canfell2

1 School of Public Health, The University of Sydney, Sydney, NSW, Australia.

2 Prince of Wales Clinical School, UNSW Australia, Sydney, NSW, Australia

**Table S1 - Subgroup size and vaccine uptake in coverage scenarios modelled**

| **Coverage scenario** | **Subgroup Interpretation** | **Included range of 3-dose coverage (%)** | **Weighted average coverage (%)** | **% population** | **Reference** |
| --- | --- | --- | --- | --- | --- |
| Lower (“USA”) | Low coverage | <25 | 23.0 | 16.2 |  |
|  | ModLow coverage | 25 to <30 | 26.3 | 28.1 | [[1](#_ENREF_1),[2](#_ENREF_2)] |
|  | ModHigh coverage | 30 to <40 | 34.7 | 38.0 |  |
|  | High coverage | 40+ | 43.6 | 17.7 |  |
| Higher (“Australia”) | Low coverage | <70 | 64.5 | 12.3 | [[3](#_ENREF_3),[4](#_ENREF_4)] |
|  | Mod coverage | 70 to <75 | 72.3 | 60.3 |  |
|  | High coverage | 75+ | 76.3 | 27.3 |  |
| Moderate (extreme inequality) | Low coverage | [exploratory scenario] | 10 | 50 | Assumption |
|  | High coverage | [exploratory scenario] | 90 | 50 | Assumption |

# **Additional Analyses Performed**

This document describes additional analyses which were done. These were:

1. Sensitivity analyses, examining model parameters previously found to have the most influence on outcomes
2. Two exploratory analyses. One was performed in order to explore whether heterogeneity in outcomes was related only to the degree of heterogeneity in uptake, or whether there was an interaction between the degree of heterogeneity in uptake and overall uptake level. The second exploratory analysis was performed in order to compare our findings with those of a previous modelled analysis of HPV vaccination in the context of unequal vaccine uptake.

# **Sensitivity Analyses**

## Methods

Parameters which were found to have the most influence on outcomes, based on prior work,[[5](#_ENREF_5)] were varied during sensitivity analysis. These parameters were vaccine coverage and aspects of vaccine efficacy. Sensitivity analyses were performed to characterise the uncertainty in predictions by varying i) vaccine coverage in boys (holding vaccine coverage in girls and duration of vaccine protection at their baseline values); and ii) duration of vaccine protection assumptions (holding vaccine coverage in females and males at baseline values). Natural history assumptions were not varied, as previous work showed that using alternative model parameter sets which had been fitted to HPV prevalence had little impact on predicted outcomes [[5](#_ENREF_5)].

Two scenarios for vaccine coverage in males relative to females were explored: in the baseline scenario, coverage in males was assumed to be equal to that in females; in sensitivity analysis we also explored a scenario where coverage in males was equal to half of that in females. Female coverage was not varied further during sensitivity analysis, as there were already three broad levels of coverage examined (lower, intermediate, higher), and the lower and higher coverage estimates were derived from observed data from Australia and the USA.

Vaccine duration was assumed to be lifelong in both males and females in the baseline analysis. During sensitivity analysis we examined the alternative assumptions of a vaccine duration of 10 years, either in both sexes, or in females only. These were chosen in order to examine scenarios where including males has previously been shown to provide the greatest incremental benefit. We did not examine a scenario where vaccine protection was better in females than in males, as previous studies have already demonstrated that assuming more favorable vaccine characteristics in females (encompassing efficacy and duration of protection) reduces the incremental impact and cost-effectiveness of including males, and particularly so when vaccine protection is assumed to be poorer in males than in females [[6-10](#_ENREF_6)]. When vaccine duration was assumed be 10 years, vaccine protection was assumed to be perfect for those 10 years, and then be lost entirely. We did not explore intermediate durations of vaccine protection (for example 20 years), or more gradual loss of protection, as these would result in outcomes which were already encompassed within the range of assumptions explored in the base case and sensitivity analyses.

## Results

We explored the impact of the duration of vaccine protection and coverage in boys on the results. Adding males to a female-only program consistently improved outcomes in all subgroups, regardless of overall coverage level, or duration of vaccine protection. The incremental benefit of adding males was greatest when vaccine protection was long-lasting in males, but not in females. Adding males also consistently increased between-group differences in outcomes compared to the between-group differences in the female-only program, however the extent of this varied (Figure 5). Scenarios where coverage was lower in males, or duration of vaccine protection was short in males, had less impact on the degree of inequality. However the reason for this was because in practice these programs were less effective over the long term, and so the relatively greater benefits experienced by the groups with higher uptake were short-lived and did not increase between-group differences over the long term.

# **Exploratory Analyses**

## The interaction between overall coverage and heterogeneity

The purpose of this analysis was to explore if the impact on outcomes of the same degree of heterogeneity varied in relation to absolute levels of coverage.

In this exploration, we re-scaled coverage in the higher and lower coverage scenarios, so that the overall population coverage was the same as in the baseline analysis, but the degree of heterogeneity in uptake was higher in the higher coverage scenario, and lower in the lower coverage scenario. Specifically, we used overall coverage based on observed Australian data but with heterogeneity in uptake based on USA data (higher coverage, higher heterogeneity scenario), and conversely overall coverage based on observed USA data but with heterogeneity in uptake based on observed Australian data (lower coverage, lower heterogeneity scenario). In the lower coverage, lower heterogeneity scenario, both the difference in outcomes between the “correlated” and “unrelated” variants and the between-group differences decreased compared to the baseline lower coverage (and higher heterogeneity) scenario. Conversely, in the higher coverage, higher heterogeneity scenario, both the difference in outcomes between “correlated” and “unrelated” variants and the between-group differences increased compared to the baseline higher coverage scenario (which had lower heterogeneity). However in both cases, the degree of inequality in outcomes was not only related to the degree of heterogeneity in vaccine uptake, as the same degree of heterogeneity in uptake resulted in different values of the pseudo Gini coefficient, depending on the level of coverage. For example, the pseudo Gini coefficient in the higher coverage, higher heterogeneity scenario was higher (indicating greater between-group differences) than in the baseline lower coverage scenario, even though the degree of heterogeneity in vaccine uptake was the same in both of these scenarios (Figure 5). This was because in the higher coverage scenario the incidence of disease in the subgroup with highest coverage was extremely small, and therefore so was the relative burden of disease, compared to other subgroups.

## Comparison with Malagón *et al*

Our analysis predicted that adding males to an existing female-only vaccination program tended to increase differences in health outcomes between the different population sub-groups modelled. However, an earlier modelled analysis by Malagón *et al* had reported that inequalities in vaccine effectiveness between the groups they modelled were reduced by inclusion of boys in the program [[11](#_ENREF_11)]. While our analysis differed from that performed in Malagón *et al*, we wished to explore these apparent differences in one of our conclusions about including males further. Broad similarities between the two analyses included that both used dynamic models of HPV infection and so took into account indirect (herd) effects. The main differences in approach were the subgroups examined (groups with differing levels of sexual behaviour [[11](#_ENREF_11)] versus groups who did not mix sexually, but did not differ in their overall activity levels), the subgroup outcomes prior to vaccination (pre-existing inequalities [[11](#_ENREF_11)] versus no pre-existing inequalities), and the degree of heterogeneity in uptake (none [[11](#_ENREF_11)] versus heterogeneity in both females and males). Another difference in approach was the aspect of the program which was used to explore equality. Our analysis considered inequalities in *disease* or *outcomes* (defined as risk of incident HPV16 infection), which was simplified by the fact that the groups had equivalent outcomes prior to vaccination. The study reported by Malagón *et al* considered inequalities in “*vaccine* *effectiveness”*, which was defined in that study as the percentage reduction in HPV16/18 prevalence at equilibrium for each group, relative to pre-vaccination levels. Specifically, a vaccine effectiveness ratio was calculated to explore inequality, using the group with the lowest level of sexual behaviour as the reference group. This ratio approach is similar to our estimate of RR_L_, except that a different measure is used to calculate the ratio (absolute risk of infections in our study, versus the percentage reduction in risk of infections in Malagón *et al*). Thus the key difference in the measures used to assess inequalities was that one used an absolute measure of the *amount of* disease in each group (outcomes; here), while one was related to the *change in* disease which was brought about as a result of the program (“vaccine effectiveness” in Malagón *et al*). There were also of course differences in the detailed sources of data and model parameters used (for example both incorporated local data on sexual behaviour and were fitted to local data). However, previous independent analyses with the two models had produced broadly comparable results, for example that around 70% of the benefits to males of a both-sex program accrue from the female-only program [[5](#_ENREF_5),[9](#_ENREF_9)]; while quantitative differences in model predictions were explicable via differences in model assumptions [[12](#_ENREF_12)]. Therefore we considered that broad conclusions from the models may be expected to be similar.

In the additional analysis we calculated a similar measure to examine inequality (“vaccine effectiveness” ratio) and modelled a more analogous coverage scenario, in order to explore these apparent differences in findings around inequalities. However, because of the differences in the factors associated with vaccine uptake examined in each study (level of sexual behaviour [[11](#_ENREF_11)] versus factors affecting partner choice here), the coverage scenario could not be exactly the same. This is because, although the study reported in Malagón *et al*, generally considered scenarios where vaccine uptake was heterogeneous (or “maldistributed”), in the specific analysis examining inclusion of males, uptake was uniform across the sexual behaviour groups, and the differences in the impact of the female-only program between groups were driven by the fact that herd effects were weaker in groups with higher levels of sexual behaviour, rather than differences in uptake [[11](#_ENREF_11)]. Because the coverage subgroups in our analysis had similar behaviour (although with variation in behaviour *within* the coverage subgroup), uniform uptake of female vaccination in the groups we examined would have resulted in no inequality between the groups, and so it was not possible to examine our primary question around subgroup outcomes of whether a female-only program with unequal outcomes in different subgroups might be made more equal by including males In order to create a female-only program with unequal outcomes, we assumed uptake was heterogeneous (“maldistributed”) in females, but then examined the impact of including males with no heterogeneity in male uptake (Figure 5).

Like Malagón *et al* we also found that in this situation inequalities in *vaccine* *effectiveness* (ie percent reduction in infections at post-vaccination equilibrium) were reduced by including males with no heterogeneity in uptake. However, we also found that there remained inequality of *outcomes* (ie incident infections) when males were included, even when there was no heterogeneity in male uptake. Furthermore, we found that inequalities in outcomes were increased compared to a female-only program; although to a lesser extent than when uptake in males was similarly heterogeneous to uptake in females (Figure 5; Table S2). This meant that in our model the benefit of the vaccination program became more similar for the subgroups (less inequality in vaccine effectiveness), but that the remaining burden of disease in the population nonetheless became more and more concentrated in subgroups with lower coverage.

Therefore, one important reason for the difference in broad conclusions around adding males with uniform uptake appears to be the outcome measure used to examine inequality – that is, whether the inequality measured relates to the *level and distribution of disease* between groups, or to the *relative impact* of vaccination. We believe that where this is possible, considering *disease* as an outcome measure is preferable to considering the *change in* disease as a result of the program. Firstly, outcomes are what is typically used in measuring inequalities and comparing health outcomes, and so are a standard measure [[13](#_ENREF_13),[14](#_ENREF_14)]. There are a range of widely used methods which may be used to examine inequalities in health outcomes [[13](#_ENREF_13),[15](#_ENREF_15),[16](#_ENREF_16)]. Also, because disease burden best represents the ultimate area of interest – the ‘end’ of the program in a sense, rather than the ‘means’. A ratio considering the relative impact of a vaccination program in different subgroups is insensitive to the remaining disease burden in a population post-vaccination, including both the absolute level of disease in the community, and the way in which this burden is distributed between subgroups. While the conclusions around whether a program increases or decreases inequality based on a “vaccine effectiveness” ratio will often coincide with conclusions based on measures relating to disease burden in a population (such as the pseudo Gini coefficient and the risk ratio, as used in our analysis), they do not always. The reason is that an estimate based on the remaining disease burden in a population post-vaccination (as employed here) will incorporate aspects of both the *relative* effect of vaccination in each subgroup (equivalent to the vaccine effectiveness ratio) and also the *absolute* effect in each subgroup. It is also likely, though, that part of the difference in the conclusions from the two studies is because the inequalities in the subgroups explored by Malagón *et al* [[11](#_ENREF_11)] were driven by greatly varying herd effects in the subgroups, since even uniform uptake in females resulted in increased inequality.

Some insight into why inequalities did not diminish in our analysis even with uniform uptake in males can again be provided by considering the probability that at least one partner in a relationship is vaccinated, which is the source of the inequality in outcomes between the groups. Where there is heterogeneity in female-only vaccination, this probability that at least one partner in a relationship is vaccinated already differs between groups, and a difference would remain in this probability even if there were uniform uptake in males. Inclusion of males could only overcome heterogeneity in females if the reverse heterogeneity occurred in males (that is if male uptake in the subgroup where female coverage is lowest was equal to female uptake in the group where this is highest, and vice versa), or uptake in males was uniform but so high that the differences between the groups effectively disappeared (however, neither of these would seem likely in a setting where there was heterogeneity in female uptake).

**Table S2 – Summary of main results in exploratory analysis, by sex, coverage scenario and program type**

|  | **Pseudo Gini coefficient** ^a^ | | **RR_L_** | | **Vaccine effectiveness ratio** | |
| --- | --- | --- | --- | --- | --- | --- |
| **Sensitivity analysis scenario** | **Females** | **Males** | **Females** | **Males** | **Females** | **Males** |
| Higher coverage (“Australia”) |  |  |  |  |  |  |
| Female-only | 0.094 | 0.058 | 1.9 | 1.5 | 0.876 | 0.801 |
| *Both sexes, male uptake heterogeneous* | *0.221* | *0.209* | *4.2* | *3.9* | *0.936* | *0.929* |
| *Both sexes, male uptake uniform* | *0.127* | *0.088* | *2.3* | *1.8* | *0.965* | *0.972* |
|  |  |  |  |  |  |  |
| Lower coverage (“USA”) |  |  |  |  |  |  |
| Female-only | 0.077 | 0.044 | 1.5 | 1.3 | 0.545 | 0.491 |
| *Both sexes, male uptake heterogeneous* | *0.126* | *0.120* | *2.1* | *2.0* | *0.597* | *0.595* |
| *Both sexes, male uptake uniform* | *0.079* | *0.045* | *1.6* | *1.3* | *0.729* | *0.827* |
|  |  |  |  |  |  |  |

RR_L_ is the risk experienced by the subgroup with the lowest vaccine coverage relative to that in the subgroup with the highest vaccine coverage, obtained by dividing the age-standardised rate of incident HPV16 infections in the subgroup with the lowest vaccine coverage by the corresponding risk in the subgroup with the highest vaccine coverage. a Theoretical maxima for pseudo Gini coefficients are 0.8766 (“Australia” scenario), 0.8378 (“USA” scenario) and 0.5 (“extreme inequality” scenario). Vaccine effectiveness ratio is based on the definition in Malagón *et al* [[11](#_ENREF_11)], and represents the long-term reduction in HPV infections in the group with the lowest vaccine uptake, relative to the group with the highest vaccine uptake.

# **References**

1. Centers for Disease Control and Prevention (2011) National, State and Local Area Vaccination Coverage among Adolescents Aged 13 through 17 Years - United States 2010. Morbidity and Mortality Weekly Report 60: 1117-1123.

2. U. S. Census Bureau (2011) Annual Estimates of the Resident Population for the United States, Regions, States, and Puerto Rico: April 1, 2010 to July 1, 2011 (NST-EST2011-01).

3. Department of Health and Ageing (2011) Human Papillomavirus (HPV): Immunise Australia program. Department of Health & Ageing.

4. Australian Bureau of Statistics (2008) Population by Age and Sex, Australian States and Territories, Jun 2002 to Jun 2007.

5. Smith MA, Lew JB, Walker RJ, Brotherton JM, Nickson C, et al. (2011) The predicted impact of HPV vaccination on male infections and male HPV-related cancers in Australia. Vaccine 29: 9112-9122.

6. Kim JJ, Goldie SJ (2009) Cost effectiveness analysis of including boys in a human papillomavirus vaccination programme in the United States. Bmj 339: b3884.

7. Chesson HW, Ekwueme DU, Saraiya M, Dunne EF, Markowitz LE (2011) The cost-effectiveness of male HPV vaccination in the United States. Vaccine 29: 8443-8450.

8. Jit M, Choi YH, Edmunds WJ (2008) Economic evaluation of human papillomavirus vaccination in the United Kingdom. Bmj 337: a769.

9. Brisson M, van de Velde N, Franco EL, Drolet M, Boily MC (2011) Incremental impact of adding boys to current human papillomavirus vaccination programs: role of herd immunity. J Infect Dis 204: 372-376.

10. Elbasha EH, Dasbach EJ (2010) Impact of vaccinating boys and men against HPV in the United States. Vaccine 28: 6858-6867.

11. Malagón T, Joumier V, Boily M-C, Van de Velde N, Drolet M, et al. (2013) The impact of differential uptake of HPV vaccine by sexual risks on health inequalities: A model-based analysis. Vaccine 31: 1740-1747.

12. van de Velde N, Brisson M, Boily MC (2010) Understanding differences in predictions of HPV vaccine effectiveness: A comparative model-based analysis. Vaccine 28: 5473-5484.

13. Spinakis A, Anastasiou G, Panousis V, Spiliopoulos K, Palaiologou S, et al. (2011) Expert Review and Proposals for Measurement of Health Inequalities in the European Union. Luxembourg.

14. Tang KK, Petrie D, Rao DS (2007) Measuring health inequalities between genders and age groups with realization of potential life years (RePLY). Bull World Health Organ 85: 681-687.

15. Asada Y (2005) A framework for measuring health inequity. J Epidemiol Community Health 59: 700-705.

16. Wagstaff A, Paci P, van Doorslaer E (1991) On the measurement of inequalities in health. Soc Sci Med 33: 545-557.
